# Optimization of a transferred arc reactor for metal nanoparticle synthesis

**DOI:** 10.1007/s11051-016-3559-y

**Published:** 2016-08-29

**Authors:** Matthias Stein, Frank Einar Kruis

**Affiliations:** Institute of Technology for Nanostructures (NST) and Center for Nanointegration Duisburg-Essen (CENIDE), University of Duisburg-Essen, Bismarckstr.81, 47057 Duisburg, Germany

**Keywords:** Metal nanoparticles, Transferred arc, Plasma synthesis, Crucible design, Production rate

## Abstract

The demand for metal nanoparticles is increasing strongly. Transferred arc synthesis is a promising process in this respect, as it shows high production rates, good quality particles and the ability of up-scaling. The influence of several process parameters on the performance of the process in terms of production rate and particle size is investigated. These parameters are the electrode design and adjustment, the gas flow rate and power input. A novel feeding mechanism allows process operation over an extended time period. It is shown that the process is capable of producing pure metal nanoparticles with variable primary particle sizes and comparatively high production rates. Optimal process conditions for a single transferred arc electrode pair are found, which allow further scale-up by numbering up.

## Introduction

Metal nanoparticles are used in numerous applications and products (Jain et al. 2007; Luo et al. 2006; Feldheim and Foss 2002). To mention just a few applications, copper nanoparticles are used to increase the thermal conductivity of nanofluids (Liu et al. 2006) and nickel particles are used for the catalytic purposes (Kim et al. 2005). The number of applications and related products is steadily increasing as well as the number of scientific reports and patents. As the industry is increasing its application of metal nanoparticles in their commercial products, the demand for high-quality metal nanoparticles is rapidly increasing. However, the state of the art does not offer a process which fully answers this increasing demand. A promising way to produce pure metal nanoparticles in larger amounts is the transferred arc synthesis (Mahoney and Andres 1995; Celik et al. 2002). It can deliver potentially a high output of nanoparticles, without using any hazardous or expensive precursors. A further advantage is the cost-effective power supply, as an off-the-shelf TIG welder can be used to ignite and maintain the arc (Chen et al. 2006).

The carrier gas composition has a marked influence on the production rate and particle size in transferred arc nanoparticle synthesis (Murphy et al. 2009). For non-nitride-forming metals, it has been found that nitrogen is by far the most efficient carrier gas (Stein et al. 2013a). This is a result of the enhanced arc power of nitrogen and a bubble formation effect resulting from molecular gas attributes. The production rates of, e.g. copper nanoparticles, nitrogen exceeds one of the copper nanoparticles in argon by several magnitudes. Reactive transferred arc synthesis can also be used, e.g. to synthesize ceramic nanoparticles (Kiesler et al. 2015).

The cost efficiency of the setup makes the process scalable by numbering up, as the TIG welder power supplies are much more affordable than dedicated high-power plasma sources. This numbering up is necessary, as an increase of production rate for a single arc by increased power input leads to too large particles (Uda et al. 1983). However, before numbering up, the basic process of one electrode pair needs to be understood well and to work as efficiently as possible. This study investigates the optimization of a single transferred arc process, which can be used then for scale-up by numbering up. Optimization is done in terms of production rate, specific electricity consumption and primary particle size, and is mainly done by means of an optimization of the crucible design as well as the process parameters plasma current and gas flow rate.

## Experimental setup

**Fig. 1:**
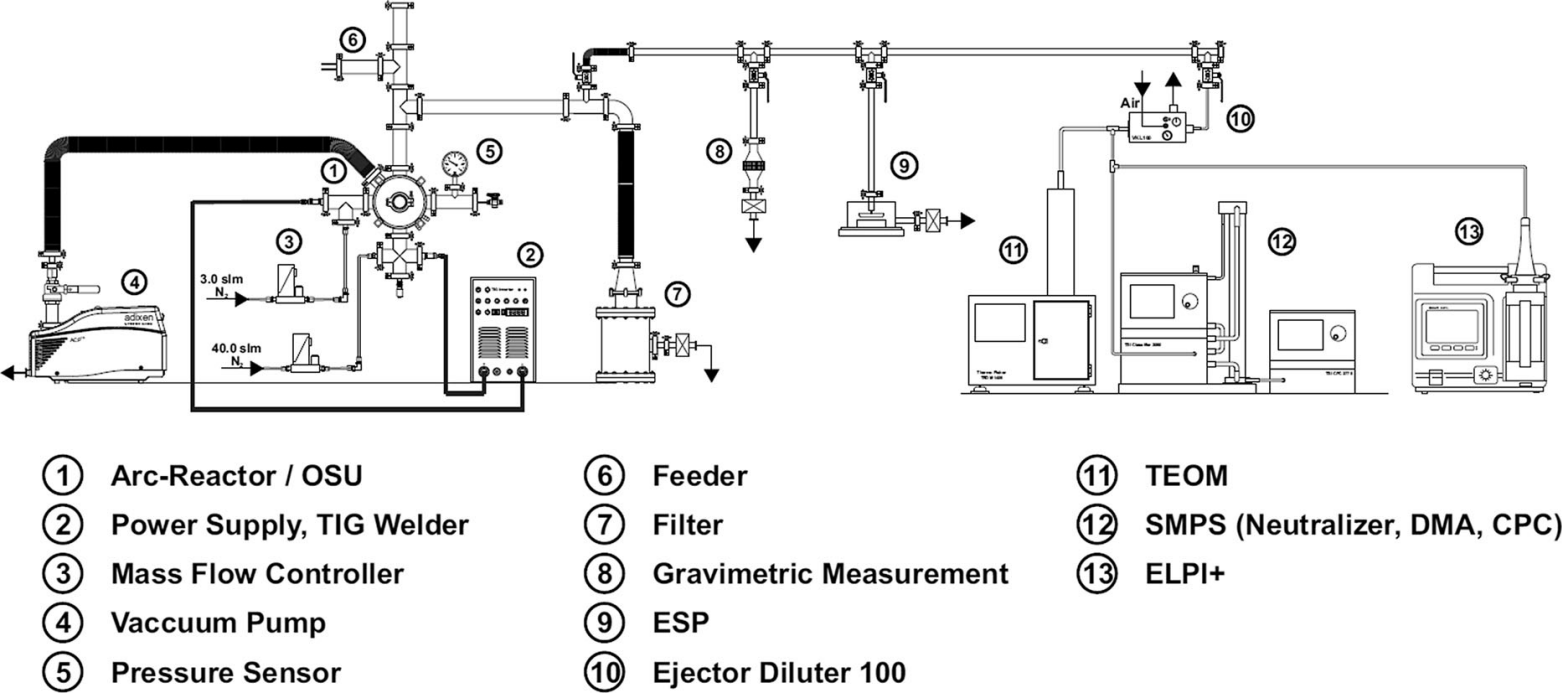
Experimental setup

Figure 1 shows the experimental setup. The heart of the setup is the reactor chamber (1), in which the particle formation takes place. The experimental setup, including the reactor chamber and its functional principle, has been discussed in more detail before (Stein et al. 2013a). For this process, patent applications have been filed (DE102014220817A1, US 2016/0101402A1, EP3009187A1).

The reactor chamber is evacuated by a vacuum pump (4) prior synthesis and refilled by two gas flows, adjusted by mass flow controllers (3). The process pressure is atmospheric. During processing, one gas flow is used as carrier gas flow (*Q*1) and the other one as a quench gas flow (*Q*2). The arc is ignited by a commercial TIG welder (2) between a tungsten rod cathode and a graphite crucible anode, which carries the material to be evaporated by the arc. The crucible serving as anode is a crucial part of the transferred arc synthesis. Multiple crucible designs and electrode arrangements are therefore tested. All crucibles are made of a massive graphite rod. A cylindrical recess with a diameter of 20 mm and a depth of 10 mm is used to fill in the feedstock material to be evaporated. On the other side, a recess with a diameter of 21 mm allows to place it on top of a tungsten rod serving as crucible carrier. The crucible is not cooled in order to minimize the energy losses and to keep the reactor chamber as simple as possible.

The aerosol formed when the metal vapour cools down is led into a filter (7) in which the particles are separated from the gas and collected as a powder. This powder can be used for BET measurement in order to determine the primary particle size of the produced particles. Before the filter, a measurement line is installed, which allows the adaption of several measurement devices to characterize further particles attributes. As measurement equipment, gravimetric measurements (8), an Electrostatic Precipitator (ESP) for microscope measurements (9), and a combination of several online measurements are arranged. A Tapered Element Oscillating Microbalance (TEOM, 11) is used to determine the aerosol mass load. A combination of Scanning Mobility Particle Sizer (SMPS, 12) and Electrical Low Pressure Impactor (ELPI, 13) is used to obtain information about agglomerate size and structure and also the primary particle size. The online measurement procedure is discussed in detail elsewhere (Stein et al. 2013b).

**Fig. 2:**
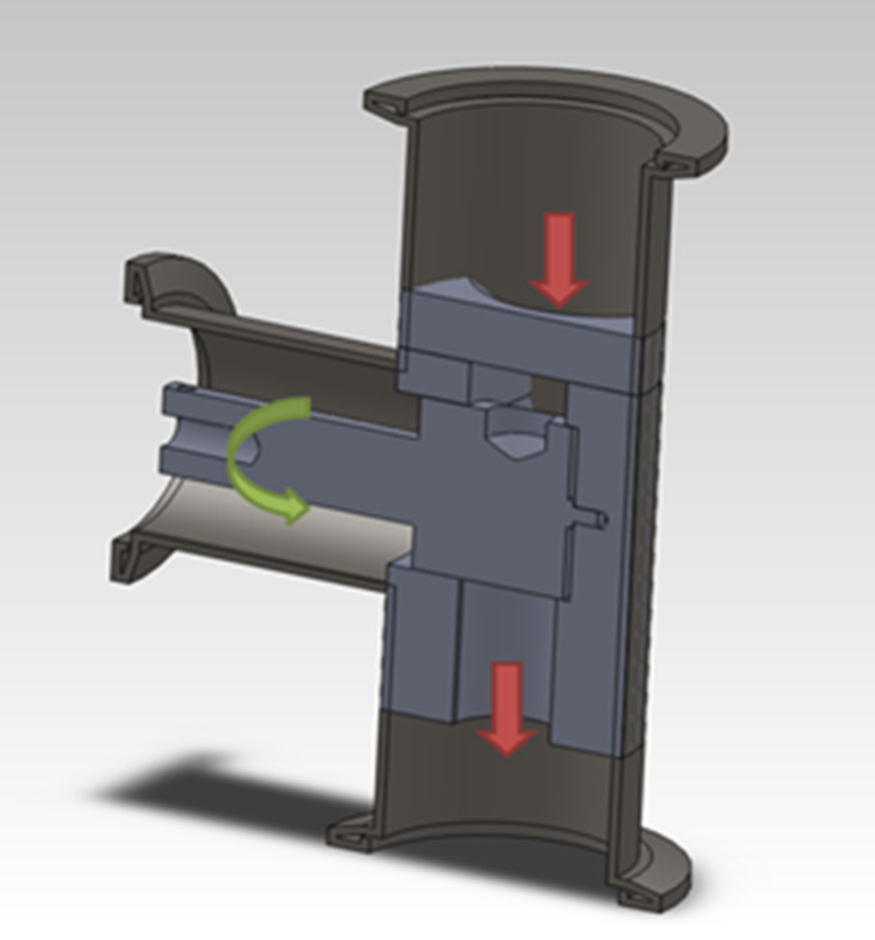
Electrically controlled feeder

At the top of the chamber, a feeding mechanism (6) is installed, which replenishes the material loss from the crucible. By keeping the crucible filled and therefore the distance between the electrodes constant, a stable production over time can be realized. The feeder is designed to refill the crucible with bulk metal with minimal vacuum leakage. Therefore, it is integrated into standard ISO-KF 40 flanges, which guarantee full vacuum capability at minimal costs. An electric motor drives a cylinder, with a small drill inside (Fig. 2). While the drill is filled with shots on the reservoir side, it is emptied on the other side, where shots drop into the crucible. It is also designed to prevent wedging of feeding material inside the mechanism and therefore can be used with a variety of forms and shapes of feedstock materials. Typically, shots of the desired metal with sizes of 1–2 mm are used. By this method, the feed amount is given by the volume of the reservoir and number of rotations, which is adjusted to the evaporation rate of the material. Here, the feeding mechanism is initiated at regular intervals accommodating the amount of material loss.

The online measurement devices are used throughout this work in order to get information about particle and agglomerate size, production rate and specific electricity consumption in dependency of different process parameters. The goal is therefore to increase the production rate, while keeping the particle size on the nanoscale. The investigated parameters and their effect on the production are the electrode arrangement and crucible design, the effect of carrier gas composition, gas flow and arc power. Furthermore, the long-term stability of the process is investigated using the dedicated feeding mechanism.

## Results and discussion

### Influence of crucible design and gas flow direction

This section describes the development of the crucible design for optimal conditions of particle synthesis via transferred arc. The reactor chamber allows due to its design multiple arrangement possibilities, allowing to test different crucible designs. The electrode arrangement allows changing the position of the two electrodes relative to each other, as well its position relative to the direction of the gas flow.

#### Electrode arrangement

**Fig. 3:**
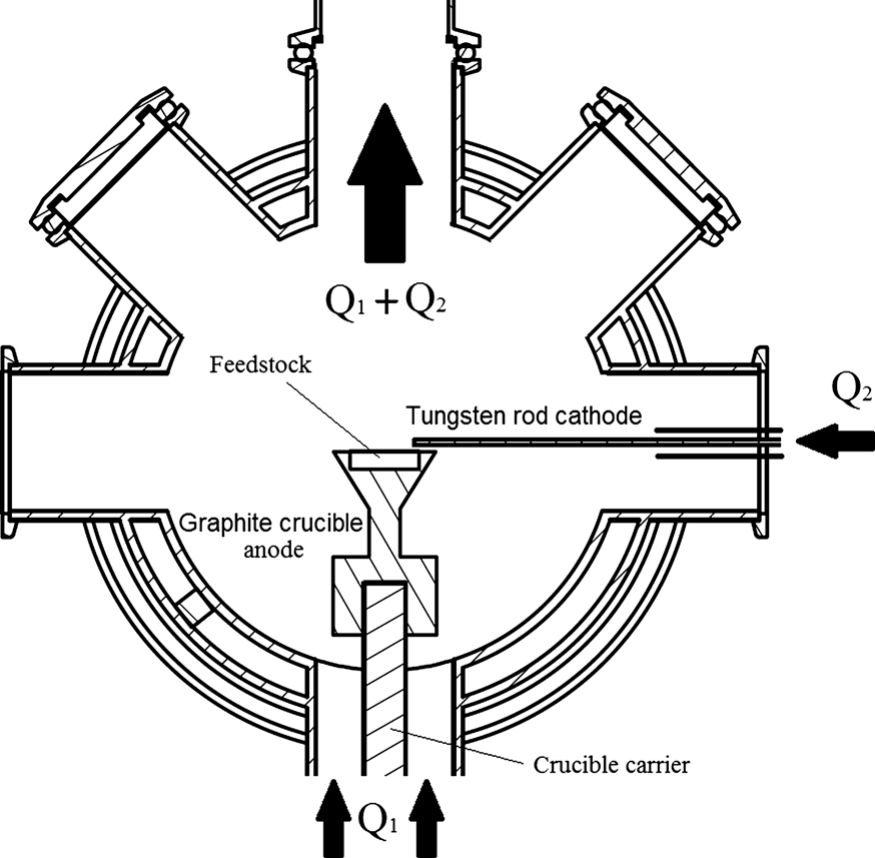
Exemplary crucible design and electrode arrangement

Figure 3 shows an exemplary electrode arrangement of the transferred arc setup. The tungsten rod cathode is placed in a 90° angle to the crucible. It has been found that this arrangement is advantageous in comparison to other positions. With this cathode position, the arc runs stable and is easy to ignite. Furthermore, high power arcs can be used without any visible cathode consumption as the arc is deflected upwards and does not heat the cathode that strongly. The crucible anode carrying the feedstock is placed on top of a crucible carrier at the bottom of the reactor chamber. Gas flows coming from different directions can be used: an axial flow *Q*1, which enters the reactor chamber at the bottom flange streaming along the crucible or a cross flow *Q*2, coming from the side streaming along the cathode directed on the arc. The aerosol outlet is the top flange of the reactor housing, unless stated otherwise.

**Fig. 4:**
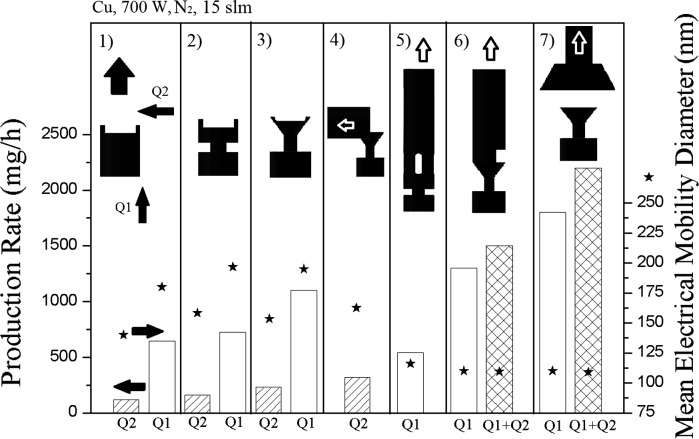
Effect of crucible design on production rate (*bars*) and particle size (*asterisks*) with *Q*1 indicating only axial gas flow, *Q*2 only cross flow and *Q*1 + *Q*2 a combination of both

Figure 4 depicts the different crucible designs and their effect on production rate (bars) and particle size (asterisks) for copper nanoparticles. Designs for open crucibles (1–3), open crucibles with guided aerosol outlet (4), guided flow crucibles (5–6) and crucibles with a funnel placed above them can be distinguished. The gas flow direction is also shown: axial flow (*Q*1), cross flow (*Q*2) or both gas flows combined (*Q*1 + *Q*2). The total gas flow (nitrogen, 15 l/min) and the applied power (35A) are kept constant.

#### Open crucibles

Crucible 1 is the simplest to manufacture and leads to a production rate of about 700 mg/h when using axial flow, the production rate in the cross flow setup (*Q*2) being much smaller. This can be explained by the fact that a gas flowing in the same direction as the buoyancy leads to less turbulences and therefore to smaller particle losses. Another advantage of the axial setup is a much more stable arc, which is especially for lower electric currents useful.

**Fig. 5:**
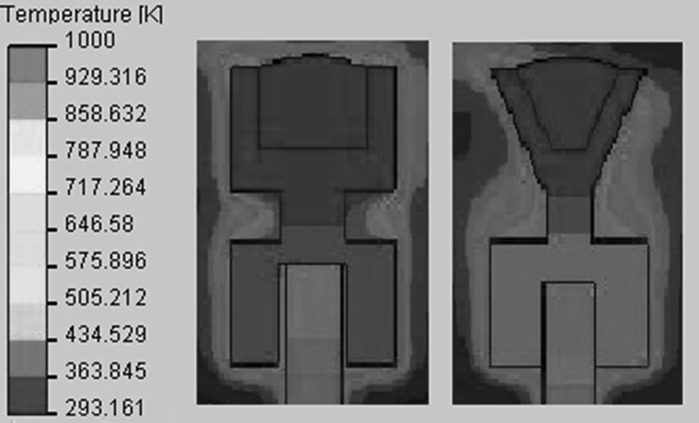
CFD simulations showing local temperatures of crucibles 2 and 3. The graphite crucible placed on top of a tungsten carrier rod

Crucible 2 has a reduced diameter in the middle part in order to reduce the heat losses towards the current feedthrough, which results in a minor increase of production rate and particle size. This approach is continued during the design of crucible 3 where the upper part of the crucible is conically shaped. Due to the decreased effective diameter, the heat losses are reduced even more, leading to a further increase in production rate. Figure 5 depicts the CFD simulations of the temperatures of crucibles 2 and 3, here it can be seen that the crucible design 3 shows lower temperatures, indicating that the heat losses to the carrier rod are reduced. With crucible 3, a production rate of copper nanoparticles above 1 g/h is achieved.

#### Open crucible with guided aerosol outlet

Further crucible designs also have a reduced effective diameter to minimize heat losses. The main goal of optimization is to reduce the particle losses inside the reactor chamber, as the water-cooled walls of the reactor are a potential thermophoretic trap for the particles formed. In crucible design 4, a graphite tube guides the flow *Q*2 out of the reactor zone in a cross flow orientation. However, the gas tends to flow upwards as a result of the buoyancy so that many particles do not enter the pipe directly. In comparison to crucible 3, a small increase in production rate is observed when using the cross flow mode (*Q*2), but the production rate is much smaller than the axial flow mode of crucible design 3.

#### Guided flow crucibles

**Fig. 6:**
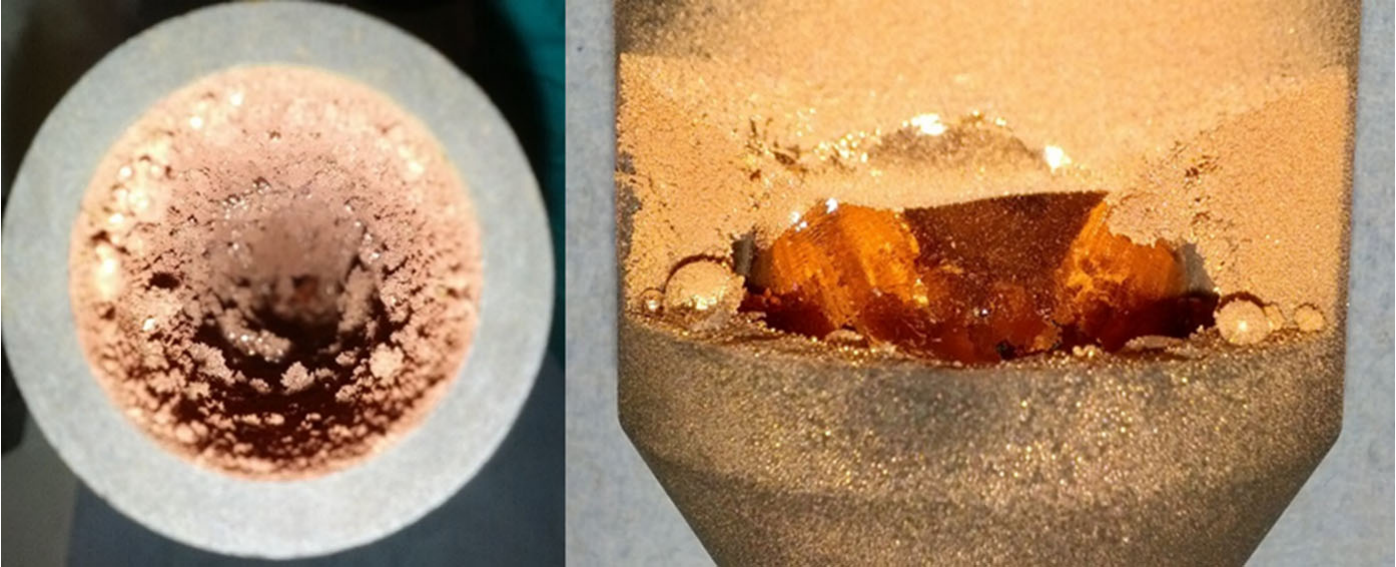
Image of crucible 6 clogged with copper particles

The axial flow mode has been used in further crucible designs, as this configuration showed in the preceding investigations to be more productive. In these designs, the aerosol flow is guided more directly out of the reactor, avoiding flow recirculation within the reactor chamber. This allows also gravity-based feeding of feedstock material through the outlet into the crucible, which is discussed in a following section. Crucible design 5 has a graphite tube with four elongated gas inlets placed above the integrated crucible which should guide the aerosol directly out of the chamber. However, the arc plasma tends to leave the graphite tube through one of the holes opposite to the cathode at higher currents. This design shows a significant decrease in production rate in comparison to the axial mode of crucible design 3 due to particles losses inside the chamber. To overcome the problem of the arc leaving the tube, the tube of crucible design 6 has only one inlet. It shows an increased production rate (1300 mg/h) at the axial flow mode (*Q*1). By adding a cross flow *Q*2 to the main flow *Q*1, the production rate is increased further, while the particle size is reduced. The combination of cross and axial flow appears to be an optimum solution to increase the production rate up to 1.5 g/h, while keeping the particle size in the desired nanoscale range. With this setup, almost no particle deposition inside the chamber is visible after synthesis. However, clogging at the inside of the crucible tube occurs after some time (Fig. 6). It seems that material splashed but not evaporated from the melt deposits inside the tube, acting also as a condensation trap for the metal vapour, so that the tube is clogged after some time. A higher gas flow rate *Q*1 (40 l/min) only leads to particle deposition closer to the melt. A further problem of this design is the thermal conductivity of the tube which results in high temperatures at the outlet flange of the reactor housing damaging the rubber sealing. Additional cooling of the flanges would require a new design of the housing and might lead to increased thermophoretic losses. Crucible design 6 is therefore only suited for low-production rate processes where the clogging does not present a problem.

#### Crucible with funnel

**Fig. 7:**
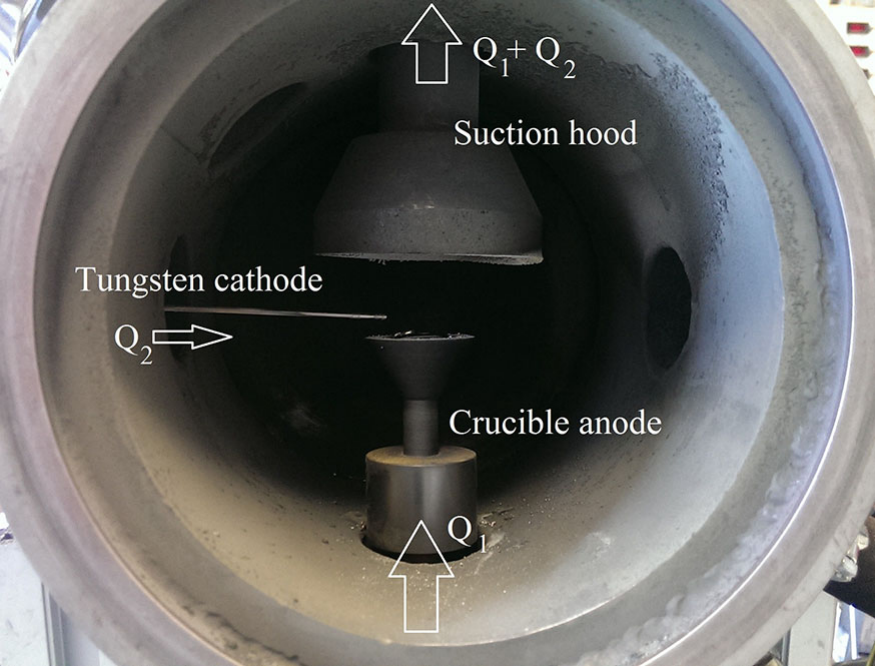
Setup with crucible design 7

**Fig. 8:**
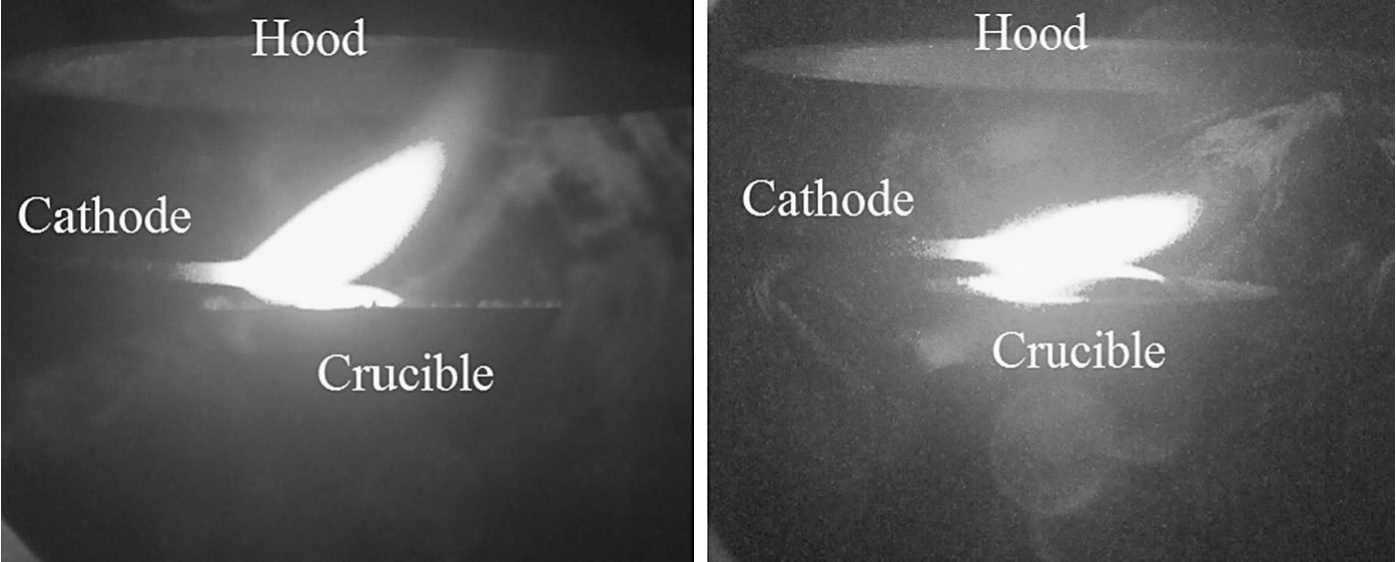
Deflection of the arc plasma due to cross flow over the cathode (*left Q*2 = 3 l/min, *right Q*2 = 5 l/min)

To surmount the clogging problem and the high temperatures of the outlet flange, in crucible design 7, the crucible is separated from the guided aerosol outlet, here a 60-mm graphite hood with an opening of 52 mm and an angle of 62°. A hood above a particle forming zone to increase the particle yield has been used, e.g. in flame reactors before (Mueller et al. 2003). Figure 7 shows the inner design of the setup with crucible design 7, which is found to result in the highest production rates and smallest primary particles sizes of all the crucible designs. It shows no clogging even after 8 h of copper nanoparticle production. This setup has several advantages in comparison with the former designs. First, clogging due to splashed particles does not occur anymore. Second, the heat losses to the outlet guidance are decreased significantly due to the separation of crucible and hood, so that production rates above 2 g/h are reached with this setup. Due to the combination of axial and cross flow, the particle size can be reduced to less than 100 nm. The cross gas flow *Q*2 has a significant influence on the primary particle size, but it also affects the arc directly. At higher values (5 l/min), it deflects the arc away from the centre (Fig. 8), but the diameter of the funnel is larger than that of the crucible so that the visible particle losses inside the reactor chamber are not so significant. Figure 8 shows a photograph of the plasma with crucible design 7 at two different cross flows. The influence of the cross flow is investigated in the following section.

### Influence of power input and gas flow on production rate and particle size

The power input has traditionally been used as a controlling parameter for material evaporation in transferred arc synthesis (Wei et al. 2006; Förster et al. 2012). The higher the power of the arc, the more energy is directed to the anode and hence to the material to be evaporated. While a higher energy input increases the temperature and therefore the evaporation of material and so the particle production rate, it increases the energy costs for the system as well.

#### Influence of power input on production rate

**Fig. 9:**
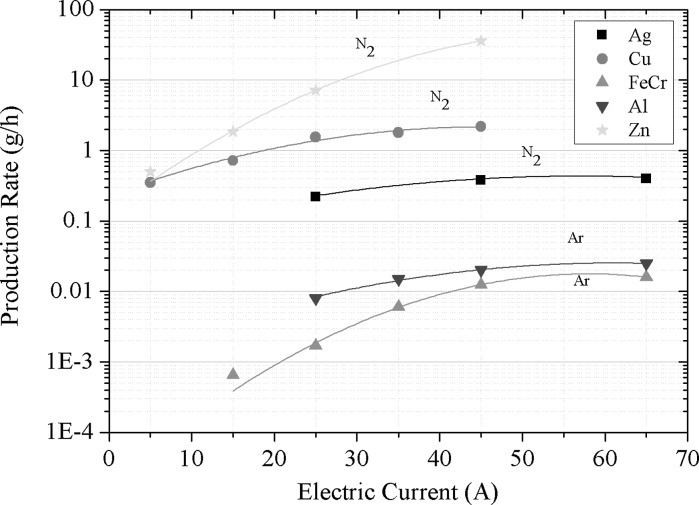
Production rate of different metals in dependence of the applied electric current (CD 6, *Q*1 = 20 l/min)

Former investigations show that a nitrogen arc has a significantly higher production rate than the other investigated arcs (Stein et al. 2013a). Therefore, for non-nitride-forming metals, nitrogen is used for the investigation of the influence of the power input and gas flow on the particle production rate. For other metals, argon is used instead. Figure 9 depicts the influence of the applied electric current on the production rate for the different metals, using crucible design (CD) 6. It can be concluded that a higher electric current leads to an increased production rate. This increase levels off at higher currents, at which a further rise in electric current does not increase the production rate. As the electric current is the controlling parameter of the power supply, the results are shown as function of current and not of power. The voltage varies only between 18 and 22 V over the range of currents used, so that the power is more or less proportional to current. The production rate is strongly material dependent, the responsible parameter for that however is difficult to determine, and is not just dependent of the materials’ vapour pressure. Other effects, such as thermal or electrical conductivity, heat flux or bubble formation can affect the production rate (Stein et al. 2013a). Of all investigated metals, zinc has the highest production rate, owned to its high vapour pressure. In nitrogen, a production rate >30 g/h has been observed at 45 A. The production rate is increased by a factor of approximately 70, by raising the applied current from 5 to 45 A. The only other metal which reaches a production rate of 1 g/h with this setup is copper. At 45 A, nearly 2.2 g/h of copper nanoparticles are produced in nitrogen. The increase of production rate in dependency of the current is not that strong in comparison to zinc.

The increase of production rate with current is the least significant for silver and aluminium. The maximum production rate of silver is determined to be around 0.4 g/h, even at 65 A, which is the maximum limit of applied current for the setup, due to the strong heat development. Aluminium is known to form nitrides; hence, argon is used as carrier gas for aluminium synthesis. At 65 A, the maximum attainable production rate is about 20 mg/h. If nitrogen is used and aluminium nitride formed, the production rate increases approximately by a factor 10. The dependency of the current stays nearly the same. FeCr shows strong dependency on the current, its production rate nevertheless is the lowest of all investigated metals. However, the feedstock composition of FeCr cannot be transformed 1:1 into aerosol particles, as the produced particle shows an excess of chromium.

#### Specific electricity consumption at different arc currents

**Fig. 10:**
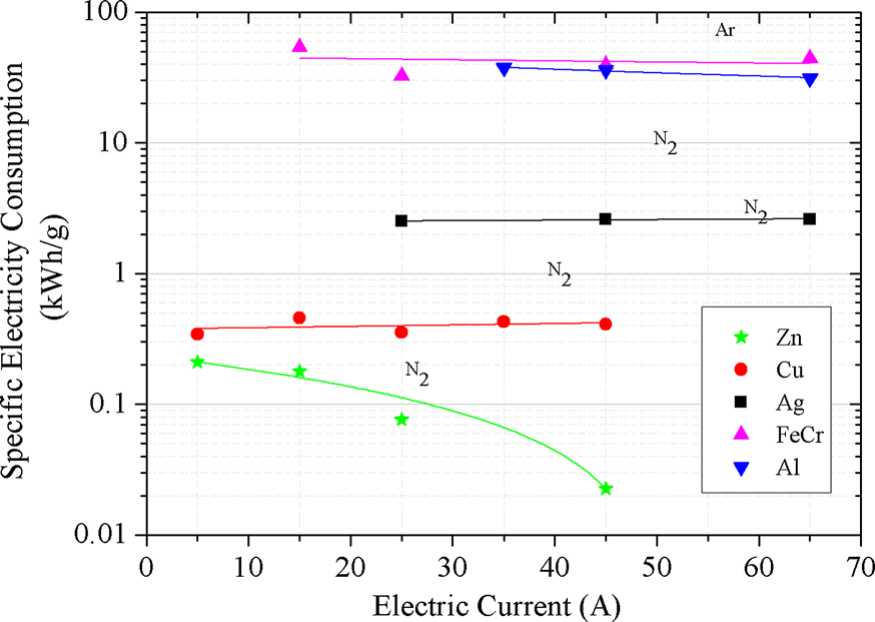
Specific electricity consumption of different metals in dependence of the applied electric current (CD 6, *Q*1 = 20 l/min)

Figure 10 shows the specific electricity consumption. Except for zinc, the specific electricity consumption is constant for all metals over the range of electric current applied. An increase in power (electric current) leads to an increase in production rate, so that the efficiency of the process stays constant, even for higher production rates. Only zinc shows a non-constant behaviour, which can be explained by strong evaporation above 5 A. At 45 A, specific electricity consumption of 25 kWh/kg is observed for zinc. As the specific electricity consumption is almost constant for the other metals in the investigated power range, it appears that the production of nanoparticles by this method can be increased by supplying more power to the process.

#### Dependence of primary particle size on arc current

**Fig. 11:**
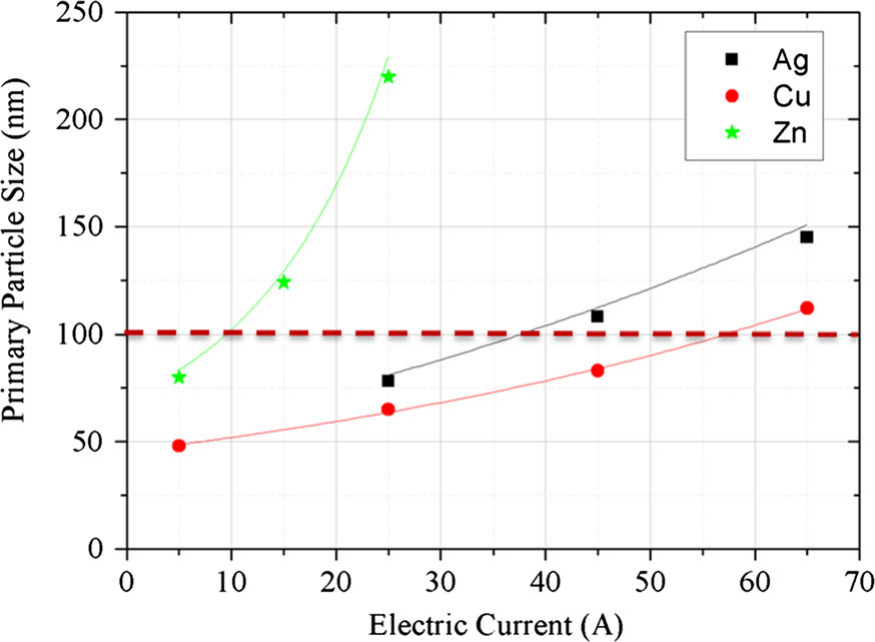
Primary particle size of different metals in dependence of the applied electric current (CD 6, *Q*1 = 20 l/min)

Figure 11 shows the primary particle size for zinc, copper, silver, and aluminium as function of the arc current (and therefore as function of input power). The biggest particles are produced with zinc, due to its high evaporation rate. The particle size ranges depending on the input current between 78 and 220 nm. Silver particles are produced in a size range of 76–144 nm (primary particle size), and copper particles between 48 and 112 nm. The particle sizes of aluminium are much smaller, as they are produced with argon as carrier gas. It is remarkable that silver exceeds the particle size of copper, although the production rate is much higher for copper than for silver. This might be consequence of the rapid sintering of silver even at low temperatures, which favours the formation of bigger primary particles and less agglomerates. However, the primary particle size increases with the applied electric current for all metals, eventually exceeding the size of 100 nm. It means by definition that no longer nanoparticles are produced. The point where this limiting value is exceeded is material dependent. Hence, an increase in production rate by simply increasing the power input has an inherent limitation.

#### Influence of gas flow rates on production rate and primary particle size

**Fig. 12:**
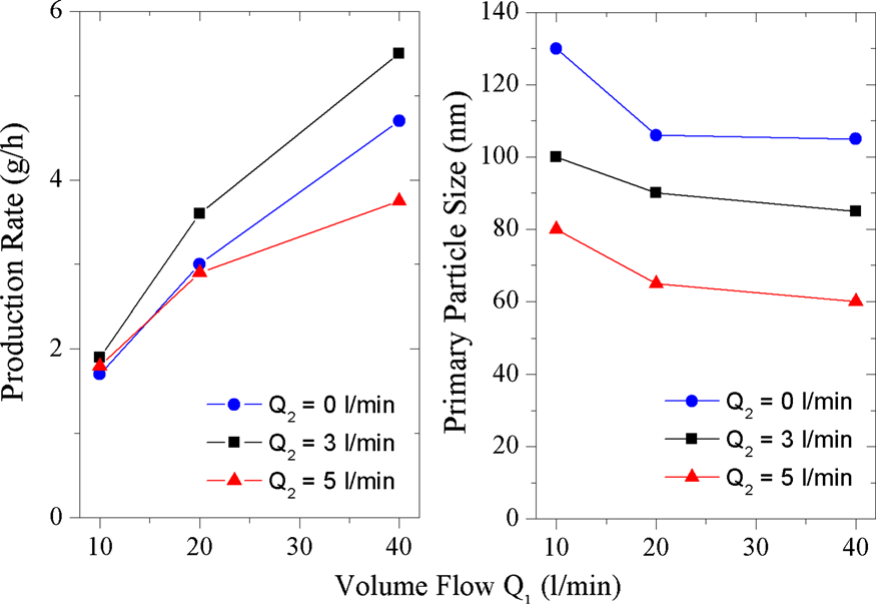
Influence of amount and direction of gas flow on production rate (*left*) and particle size (*right*) at 35 A (CD 7)

Further optimization of production rate in the nanoscale size range can be realized by the amount and direction of the gas flow, using CD 7. Figure 12 depicts the influence of the amount and direction of the gas flow on the production rate and primary particle size for copper particles at 35 A. It shows that the production rate increases with the carrier gas flow *Q*1. An explanation of this is that at higher gas flows, a more efficient transport of particles out of the formation zone occurs. Also particle losses in the lines are decreased extensively at higher gas flows. Nevertheless, volume flow higher than 40 l/min is not used due to instabilities of the arc and excessive carrier gas consumption. The second (cross) flow *Q*2 is also beneficial in terms of production rate. By adding 3 l/min from the side, the production rate can be increased about 1 g/h (*Q*1 = 40 l/min), to a value of approximately 5.5 g/h. If the cross flow *Q*2 increased further (dotted line), the production rate decreases significantly. On the one hand, the arc gets unstable at a cross flow = 5 l/min so that the production process is quite discontinuous. On the other hand, much smaller particles are formed at this flow. The particle size decreases significantly with the cross flow *Q*2, as it directly influences the residence time in the nucleation and coagulation zone. The primary particle size is the result of coagulation between particles (either spherical or agglomerated) and sintering within agglomerates if the local temperature is below the melting point. Higher gas flow rates will lead to shorter residence times, reducing not only the time molten particles that might grow due to Brownian collisions, but also reducing the time primary particles that can increase their size due to solid-state diffusion or other sinter mechanisms. The increased gas flow might also decrease the total arc temperature and hence the effective zone in which the primary particle can increase their size due to the aforementioned mechanisms. Principally, one can adjust the primary particles size of the copper nanoparticles in the range from 60 to 130 nm by varying the nitrogen gas flow. In order to reach the maximal production rate of metal nanoparticles, a main carrier gas flow *Q*1 as high as possible and a cross flow *Q*2 at about 3 l/min appear to be optimal.

#### Optimal process parameters

Table 1 summarizes the optimal parameters to produce metal nanoparticles with the present reactor in larger amounts keeping the particle size below 100 nm (except for zinc). The applied parameters are strongly material- and carrier gas-dependent. For most metals, CD7 appears to be the best in terms of production rate, which might be a consequence of the higher carrier gas flows, which can be used with this setup. The mass load per cubic centimetre stays almost constant, when the gas flow is doubled, but the particle size decreases. For aluminium and FeCr, the argon arcs with CD 6 seem to be most promising. The production rate is comparatively low due to the use of argon in comparison to the other metals. A further increase in gas flow does not lead to an increased production rate. In comparison to CD 7, CD 6 shows slightly decreased particle losses to the reactor walls and is hence preferred for metals with lower production rates. A cross flow *Q*2 is advantageous for the synthesis with CD 7. At a value of 3 l/min, the arc remains stable, while significantly smaller particles can be produced. The particle size of argon produced particles is much smaller, so that an additional decrease of particle size is not required. As discussed before, the applied electric current impacts the production rate and hence particle size strongly, which means that the highest current is not automatically the optimal process condition. The evaporation rates of copper and zinc are very high even at lower currents. An increase in current might lead to a marginal increase in evaporation and production rate, but it is not beneficial in terms of electricity consumption and particle size. Due to the heat development at higher currents, current values above 60 A have not been used.

**Table 1:** Optimal parameters for different metals with the transferred arc reactor

Metals	Carrier gas	CD	*Q*1 (l/min)	*Q*2 (l/min)	Elect. current (A)
Zinc	Nitrogen	7	40	3	25
Copper	Nitrogen	7	40	3	35
Silver	Nitrogen	7	40	3	60
Aluminium	Nitrogen	6	20	0	60
FeCr	Nitrogen	6	20	0	60

Table 2 shows the production rate, specific electricity consumption, mobility size (agglomerates) and primary particle size (BET) of the different metals achieved with the optimal parameters of Table 1. Due to its vapour pressure, zinc is the material with the highest production rate and hence lowest specific electricity consumption. More than 30 g/h of zinc nanoparticles with a mean primary particle size of 180 nm can be produced. By the techniques discussed before, the particle size can be reduced, but at the expense of decreased production rate. Copper nanopowder with a mean primary particle size of 83 nm can be produced with a rate of 5.5 g/h. The specific electricity consumption of copper production is approx. 180 kWh/kg. The maximum realized production rate of silver has been approx. 1 g/h with a particle size of 120 nm. The particle size at this production rate seems rather large, especially when compared to the values of copper. Also, the mobility size of the produced silver agglomerates almost does not differ from the primary particle size, which indicates the formation of very dense agglomerates consisting of only few, but larger particles due to sintering. The production rates of aluminium and FeCr are low, because argon has been used. The production rates are in the milligram range (30 mg/h for aluminium, 20 mg/h for FeCr and chromium). The specific electricity consumptions are hence significantly higher than the ones produced by a nitrogen arc. However, the particle sizes are very small, which can make this synthesis procedure relevant for some applications even at these low-production rates.

**Table 2:** Production rate (PR), specific electricity consumption (SEC), agglomerate mobility diameter *d*_m_ and primary particle diameter *d*_P_ of the different metals achieved with the optimal parameters from Table 1

Metals	PR (g/h)	SEC (kWh/kg)	*d*_m_ (nm)	*d*_P_ (nm)
Zinc	36	12	212	180
Copper	5.5	179	144	83
Silver	1	1139	128	120
Aluminium	0.03	32,025	65	15
FeCr	0.02	41,017	63	18

### Crucible feeding and long-term process stability

**Fig. 13:**
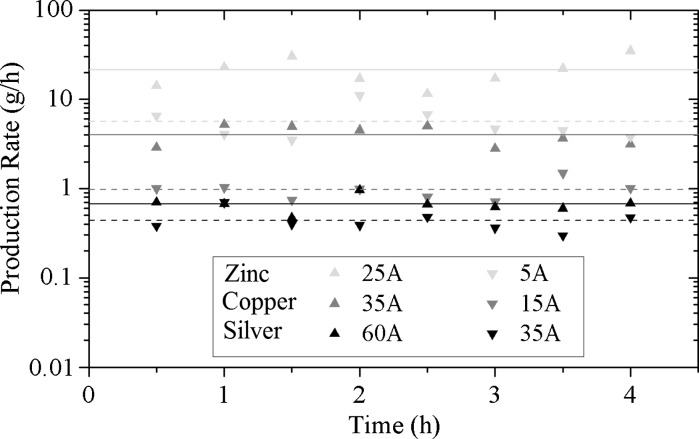
Production rate over time for different metals

In order to ensure that the process is indeed energy- and cost-efficient, the system has to run as long as possible with the minimal possible number of interruptions and operator actions. This section reports on the long-term stability of the process, using a dedicated crucible feeding mechanism, which fills the evaporated amount of metal back into the crucible. The production rate of the different metals at different electric currents, determined by gravimetric measurement, using CD 7 is plotted over a time span of 4 h as shown in Fig. 13. Measurements are performed every 30 min during an interval of 90 s. As can be seen, the production rate remains constant over the 4-h time span. Longer runs (8 h) were also possible and ran without any problems. BET measurements show constant values of primary particle sizes over the 4-h time span. The mean production rates over time differ from the maximum values determined in the previous section. The mean production rate of copper over time is with 4 g/h almost 28 % below the production rate reported earlier in this work, a possible explanation might be the lower mean level of the melt over time as compared to using a freshly filled crucible.

## Conclusion

The production rate and particle size in transferred arc synthesis are optimized by a variation of reactor design and process conditions in comparison to a previous setup. By optimizing the crucible design, an increase of production rate by a factor of 4 as compared to the earlier reported results (Stein et al. 2013a) has been realized. The increase of production rate is a result of a minimization of the heat losses at the crucible surface and therefore an increase in evaporation rate and the minimization of particle losses to the reactor chamber due to a guided aerosol outlet. The optimization of the crucible design allows furthermore an increase of carrier and quench gas flows, which results in a better control and reduction of particle size. It has been found that an increase of carrier gas flow does not only lead to a reduced primary particle size, but also to an increase of effective production rate. This increase is a result of the enhanced transport of material out of the particle formation zone. It is well known that the increase of power input results in an increase of production rate in arc synthesis; however, in this study, also a direct relation between production rate and particle size is given. It shows that there is a limit to increasing the production rate of the nanoparticles by increasing the power input. Long-term process stability has been obtained by the use of a dedicated feeding mechanism. Process runs of up to 8 h are achieved. Summarizing, it can be said that the performance in terms of production rate and particle size of a single electrode pair in transferred arc synthesis can be raised significantly. The limiting factor of production rate however is the primary particle size, which will also increase with production rate. Further increase of production rate of nanoparticles not larger than 100 nm can only be achieved by numbering up the optimized electrode pairs.
